# How orthogonal are the OBO Foundry ontologies?

**DOI:** 10.1186/2041-1480-2-S2-S2

**Published:** 2011-05-17

**Authors:** Amir Ghazvinian, Natalya F Noy, Mark A Musen

**Affiliations:** 1 Stanford Center for Biomedical Informatics Research, Stanford University, 251 Campus Drive, Stanford, CA, USA

## Abstract

**Background:**

Ontologies in biomedicine facilitate information integration, data exchange, search and query of biomedical data, and other critical knowledge-intensive tasks. The OBO Foundry is a collaborative effort to establish a set of principles for ontology development with the eventual goal of creating a set of interoperable reference ontologies in the domain of biomedicine. One of the key requirements to achieve this goal is to ensure that ontology developers reuse term definitions that others have already created rather than create their own definitions, thereby making the ontologies orthogonal.

**Methods:**

We used a simple lexical algorithm to analyze the extent to which the set of OBO Foundry candidate ontologies identified from September 2009 to September 2010 conforms to this vision. Specifically, we analyzed (1) the level of explicit term reuse in this set of ontologies, (2) the level of overlap, where two ontologies define similar terms independently, and (3) how the levels of reuse and overlap changed during the course of this year.

**Results:**

We found that 30% of the ontologies reuse terms from other Foundry candidates and 96% of the candidate ontologies contain terms that overlap with terms from the other ontologies. We found that while term reuse increased among the ontologies between September 2009 and September 2010, the level of overlap among the ontologies remained relatively constant. Additionally, we analyzed the six ontologies announced as OBO Foundry members on March 5, 2010, and identified that the level of overlap was extremely low, but, notably, so was the level of term reuse.

**Conclusions:**

We have created a prototype web application that allows OBO Foundry ontology developers to see which classes from their ontologies overlap with classes from other ontologies in the OBO Foundry (http://obomap.bioontology.org). From our analysis, we conclude that while the OBO Foundry has made significant progress toward orthogonality during the period of this study through increased adoption of explicit term reuse, a large amount of overlap remains among these ontologies. Furthermore, the characteristics of the identified overlap, such as the terms it comprises and its distribution among the ontologies, indicate that the achieving orthogonality will be exceptionally difficult, if not impossible.

## Introduction

Ontologies in biomedicine facilitate information integration, data exchange, search and query of biomedical data, and other critical knowledge-intensive tasks [1,2]. The OBO Foundry is an initiative to create a set of well-defined reference ontologies that are designed to work with one another to form a single, non-redundant system [3]. The OBO Foundry consortium defines a number of principles for ontology development and ontology developers that want their ontologies to be members of the OBO Foundry must work to conform to these principles. The principles include, for example, the requirement that the ontology is openly available; that the ontologies can be expressed in a common shared syntax; that all the terms in the ontologies have well-formed definitions; and that the ontology has the plurality of users. Ontology developers who request to have their ontology as an OBO Foundry candidate are expected to work with the OBO Foundry custodians to ensure that their ontology conforms to the OBO Foundry principles. The set of ontologies in the OBO Foundry evolves constantly. New ontologies submitted to the OBO Foundry are added to the list of candidate ontologies and the OBO Foundry community works together to bring these ontologies as close to satisfying the OBO Foundry principles as possible. After the custodians decide that an ontology conforms sufficiently to the OBO Foundry principles, it may become a bona fide OBO Foundry member.

One of the key—and, arguably, more controversial—aims of the OBO Foundry effort is to create a set of **orthogonal** ontologies, which means that each term is defined in only one ontology. Other ontologies that need to use the term refer to its definition in the source ontology. For example, the Cell Type ontology may define a term *Cell*. Then other ontologies, such as the Foundational Model of Anatomy (FMA) [4], that need to refer to the term *Cell* will refer to the the term *Cell* in the Cell Type ontology.

Thus, in order to increase orthogonality, ontology authors reuse terms from other ontologies. Each term in an OBO Foundry ontology has a unique identifier (id). The id consists of a prefix, which is the ontology abbreviation, and the id for that term within the ontology. For example, the id for the class *Biological process* in the Gene Ontology (GO) [13] is *GO:0008150*. Because each term in the OBO Foundry has a unique id (another OBO Foundry principle), the FMA can refer to the id of the term *Cell* in the Cell Type ontology whenever the FMA authors need the term *Cell*. An alternative form of term reuse for ontologies in the OBO format (currently the most common format in the OBO Foundry) is to use the *xref* property to indicate that the term that the authors are defining is a term from another ontology. While the authors still create a new term in this case—and link it to a term in another ontology—we can still consider such a reference as reuse: The authors have clearly identified the correspondence, and it can be a mechanical task to replace the newly defined class with the one that it references.

**Figure 1:**
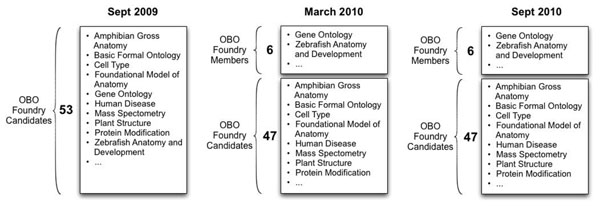
**Snapshots of the OBO Foundry ontologies for September 2009, March 2010, and September 2010.** We analyzed 53 OBO Foundry ontologies at the three dates indicated in the figure. The numbers in bold next to each group of ontologies represent the number of ontologies in the group. Several ontologies are omitted from each snapshot for readability. Beginning in March 2010, the OBO Foundry designated six ontologies as OBO Foundry members, which we analyzed separately in addition to analyzing as part of the set of OBO Foundry ontologies.

Our goal in this paper is twofold: (1) to analyze how orthogonal the OBO Foundry ontologies actually are; and (2) to analyze how the level of orthogonality in this set of ontologies has evolved over time, as the OBO Foundry community has worked to bring these ontologies closer to its stated ideal. Thus, we want to test the hypothesis that the ontologies developed by the OBO Foundry community are indeed approaching orthogonality, as the contributors work together to eliminate the overlap between the ontologies and to replace it with the reuse of terms across ontologies. Furthermore, evaluating the conformance of these ontologies to the criterion of orthogonality can serve as a measure of quality control for the OBO Foundry. We took three snapshots of the OBO Foundry candidates, each six months apart, and analyzed the extent to which this set of ontologies was approaching the goal of orthogonality. We analyzed the OBO Foundry candidates in September 2009, March 2010, and again in September 2010 (Figure 1). Our analysis examines 53 ontologies that were OBO Foundry candidates on September 1, 2009, the date that we took the first snapshot. Because many of these ontologies were originally developed independently of one another, there is a significant level of overlap among them. The OBO Foundry community states that it works actively to eliminate this overlap. Since the OBO Foundry project evolves continuously, our analysis of the data on any specific date is naturally only a snapshot of its state at that time. On March 5th, 2010, the OBO Foundry reorganized its set of ontologies, inducting the first six candidates to become OBO Foundry members [5]. This announcement asserted that these ontologies conform sufficiently to the OBO Foundry principles. We have included in this paper a separate analysis of these six ontologies.

Until now, there has been no quantitative analysis either of the level of overlap or of the level of term reuse. There is also no comprehensive list of the terms that are represented in more than one ontology and that must be reconciled. In this paper, we use a simple lexical method to find correspondences (both reused terms and overlapping terms) between terms in different ontologies. We have shown previously that this method produces good results for mapping terms in biomedical ontologies [6,7]. We use the results of this mapping to analyze the level of term reuse and term overlap in the OBO Foundry ontologies and to analyze the dynamics of these links over time.

Specifically, this paper makes the following contributions:

• We analyze the dynamics of term reuse among OBO Foundry candidate ontologies.

• We analyze the dynamics of overlap among the OBO Foundry candidates and thus quantify the amount of work that needs to be done to achieve orthogonality among the ontologies.

• We provide a list of current overlapping terms for OBO Foundry candidates, thus facilitating progress toward orthogonality.

• We analyze the level of reuse and overlap for the six ontologies that became the first OBO Foundry members.

## Methods

In each of three snapshots, we analyzed 53 ontologies that were identified as OBO Foundry candidates. These 53 ontologies contained 272,168 terms in September 2009, 311,351 terms in March 2010, and 318,872 terms in September 2010.

To perform our analysis, we processed the ontologies from the OBO Foundry repository, created lexical mappings between terms based on the terms’ preferred names, and then divided this set of mappings into the cases that constitute term reuse and the cases that constitute overlap of terms.

### Collecting and processing the ontologies

BioPortal is an ontology library that, among other features, provides browsing and visualization for more than 200 biomedical ontologies [8,9]. The BioPortal ontology library includes ontologies that individual investigators submit directly to BioPortal, as well as terminologies drawn from both the Unified Medical Language System (UMLS) [10,11] and the WHO Family of International Classifications (WHO-FIC) [12]. The BioPortal repository also includes the ontologies that are candidates or members of the OBO Foundry. BioPortal processes all the ontologies, creating a unified and easily accessible index of all the content. We used this index to perform our analysis of the ontologies that were listed as OBO Foundry candidates or members at the times of our snapshots. We excluded two ontologies from our analysis—a “timed” and an “abstract” version of the Human developmental anatomy ontology; these ontologies contain multiple classes with the same preferred name. Thus, a term from another ontology with that preferred name may map to multiple classes in one of these ontologies. Furthermore, the large inherent overlap between these two ontologies generated more than 100,000 inter-ontology mappings, with each mapped term from the “abstract” version overlapping with several terms from the “timed” version.

We analyzed separately the six ontologies that were announced as the first members of the OBO Foundry on March 5, 2010: Chemical Entities of Biological Interest (CHEBI), Gene Ontology (GO), Phenotypic Quality Ontology (PATO), Protein Ontology (PRO), Xenopus Anatomy Ontology (XAO), Zebrafish Anatomy Ontology (ZFA). The announcement asserted that these ontologies have satisfied the OBO Foundry principles to an acceptable degree and are therefore the OBO Foundry community recommends that these ontologies “serve as preferred targets for community convergence.”

### Creating lexical mappings

We used lexical mappings between ontology terms to identify cases of term reuse and to estimate the level of overlap between the ontologies.

We used the following steps to create lexical mappings between terms:

1. generate *an index of labels* that are used as preferred names of terms;

2. *normalize* the strings that represent the labels in the index;

3. find pairs of *matching* labels by comparing the normalized strings;

4. create *mappings* between terms in the ontologies based on the matching labels that we identified in the previous step.

After generating a database of labels used as preferred names of terms, we normalized all the names by converting them to lower case and removing all delimiters (e.g., spaces, underscores, parentheses). We used a MySQL database to index each label along with the ID for the ontology and the term that the label came from.

To find matching terms, we performed an SQL query on the table of normalized labels to identify pairs of strings that matched exactly. To improve precision, we compared only strings with at least three characters. We created a mapping between every pair of ontology terms where the normalized strings representing their preferred names were equal.

### Identifying reuse

We used the mappings between ontology terms to identify the level of term reuse across ontologies by employing the following constructs to identify explicit reuse of terms from one ontology by another:

Case 1: The ids of the terms were the same.

Case 2: The ids of the terms appear as if they were intended to be the same

Case 3: One term contained a reference to the id of the other term as *xref*.

Recall that the goal of the OBO Foundry is to create a set of orthogonal ontologies, where ontology developers reuse terms from other ontologies rather than define their own. The two recognized ways to reuse terms are for one ontology to refer explicitly to a term id from another ontology (Case 1) and for one ontology to use the *xref* property in the OBO format to indicate that a term is defined in another ontology (Case 3).

We also observed—and included in our count of the reused terms—cases of *intended* reuse: an ontology author uses an id for a term incorrectly, but it seems clear what the intention of the author was. For instance, because some ontologies changed their prefix over time, some reused terms use prefixes from the older versions, which are no longer valid. Similarly, there are inconsistencies in the reuse of the terms from the Basic Formal Ontology (BFO)—perhaps because BFO includes several prefixes in its term ids (*span:*, *snap:*, and *bfo*). Additionally, we identified cases where the ids were intended to refer to a term in another ontology, but were not used correctly. For example, *MP:MP_0002216* from Suggested Ontology for Pharmacogenomics (SOPHARM) is not a correct reference to the term *MP:0002216* from Mammalian phenotype (MP), but the SOPHARM authors apparently intended this id to refer to a term from the MP ontology.

The mappings that we generated automatically directly provided measures for Cases 1 and 2 in the list above. We processed the files in OBO format separately to analyze reuse through *xref*.

### Identifying overlap

Our lexical-mapping method produced pairs of terms with matching preferred names. By removing the terms that constitute reuse, we arrived at the set of terms that constitute overlap: these are terms with matching preferred names that do not refer to each other explicitly.

## Results

Applying the lexical mapping technique resulted in 20,570 mappings among the OBO Foundry candidate ontologies in the September 2009 snapshot, 22,924 mappings in the March 2010 snapshot, and 27,059 mappings in the September 2010 snapshot. In each case, we analyzed the set of reused terms and overlapping terms separately (Table 1).

**Table 1:** Summary of overlap and reuse statistics over time.

	September 2009	March 2010	September 2010
Total terms	272,168	311,351	318,872
Reused terms	10,972 (4.0%)	13,458 (4.3%)	17,067 (5.4%)
Overlap terms	9,598 (3.5%)	9,566 (3.1%)	9,992 (3.1%)

The table shows the number of terms, and the number of overlapping and reused terms for the three snapshots: September 2009, March 2010, September 2010

We then analyzed reused and overlapping terms among the six OBO Foundry members announced on March 5, 2010.

### Dynamics of reuse

**Figure 2:**
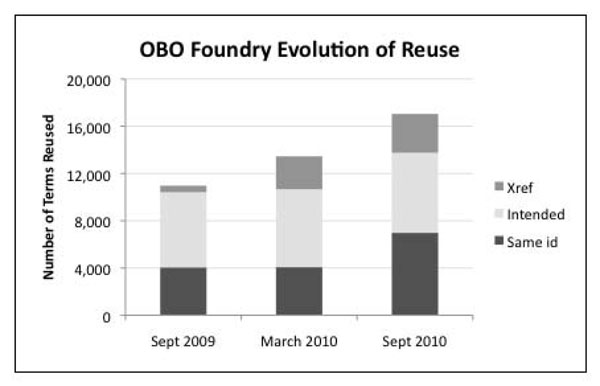
**The reuse relationships for each of our OBO Foundry candidate snapshots, broken up by type of reuse.** The height of each bar indicates the total number of reused terms for a particular snapshot. Each bar also shows what portion of the total reuse for that snapshot consists of each of the three types of reuse that we identify.

Among the 53 OBO Foundry candidates that we analyzed, reuse increased from 10,972 cases in September 2009 to 17,067 cases in September 2010. These reuse relationships comprise the three forms identified earlier. Figure 2 shows their distribution for each of our three snapshots. In each snapshot, the majority of reuse results from a term from one ontology directly referencing the id of a term from another ontology or apparently intending to do so. The smallest portion of reuse comes through use of the *xref* property.

**Figure 3:**
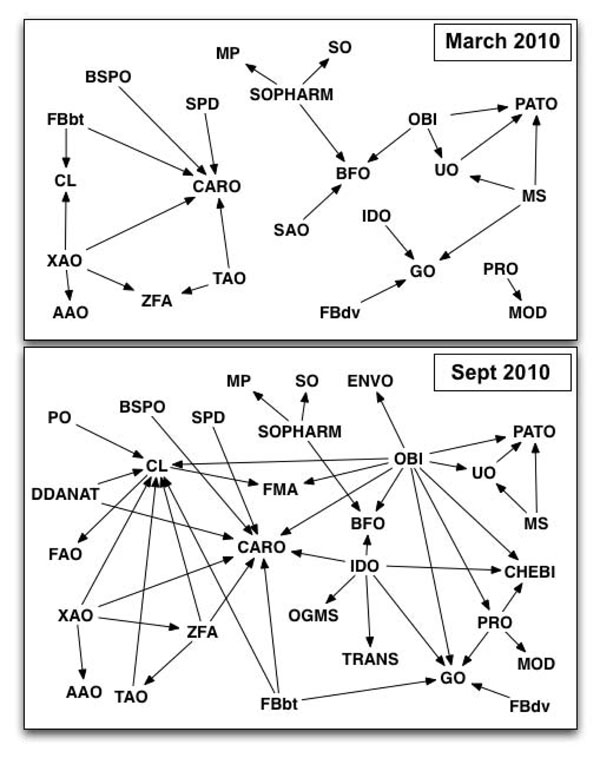
**The reuse relationships of OBO Foundry candidate ontologies for March 2010 and September 2010.** Each node represents an ontology. Please refer to http://www.obofoundry.org for the list of full ontology names corresponding to the acronyms that we use as labels in the graph. In each graph, an edge from a node representing an ontology *O*_1_ to a node representing an ontology *O*_2_ means that ontology *O*_1_ reuses at least one term from *O*_2_.

We constructed graphs showing reuse relationships among ontologies for the March and September 2010 snapshots (Figure 3). In each graph, an arrow from a node representing one ontology to a node representing another ontology means that the former reuses some terms from the latter. The figure illustrates both the current state of reuse and the change in reuse among ontologies over the six month period from March 2010 to September 2010.

Currently, 16 of the 53 ontologies (30%) reuse at least one term from another ontology and 19 ontologies (36%) have at least one of their terms reused. The Common Anatomy Reference Ontology (CARO) and the Cell Type ontology (CL) are the two most commonly reused ontologies; they have 8 and 7 ontologies reusing terms from them respectively. Notably, as of September 2010, CARO had three more ontologies reusing terms from it than it did in March.

The Ontology for Biomedical Investigations (OBI) and the Infectious Disease ontology (IDO) reuse terms from the greatest number of ontologies: OBI reuses terms from 10 different ontologies and IDO reuses terms from 6 ontologies. IDO reuses a total of 50 terms (or 11% of its total terms) and OBI reuses 177 terms, about 7% of its total.

Overall, if we define a reuse relationship to be a relationship between two ontologies where one ontology reuses at least one term from the other, the number of reuse relationships among the 53 OBO Foundry candidate ontologies almost doubled between March and September 2010, increasing from 24 to 45. In addition to analyzing the patterns of reuse among ontologies, we examined the extent to which each of these ontologies reuses terms from other ontologies. We found that 16 ontologies reuse at least 10% of their terms. Table 2 shows, for each of these ontologies, the number of terms that the ontology reuses, relative to the size of the ontology.

**Table 2:** The extent of reuse for each OBO Foundry candidate that reuses at least 10% of its terms from other ontologies.

Ontology	Reuses From	# of Terms Reused	Ontology Size	% Reused
ZFA	CARO, TAO, CL	2,505	2,593	96%
UO	PATO	2,150	2,425	88%
SOPHARM	BFO, MP, SO	6,624	8,603	77%
MS	PATO, UO	2,417	3,887	62%
XAO	AAO, CARO, ZFA, CL	262	817	32%
TAO	CL, CARO	402	3,001	13%
IDO	BFO, GO, OGMS, CHEBI, CARO, TRANS	50	449	11%

Column (1) is the ontology id; (2) is the set of ontologies from which it reuses terms; (3) is the number of terms reused; (4) is the number of terms in the ontology; (5) is the percent of the ontology is composed of reused terms.

### Dynamics of overlap

From lexical mapping, we found that the number of overlaps among the OBO Foundry candidates increased slightly from 9,598 in September 2009 to 9,992 overlaps in September 2010. In the most recent snapshot, 51 ontologies (96%) have at least one overlapping term.

**Figure 4:**
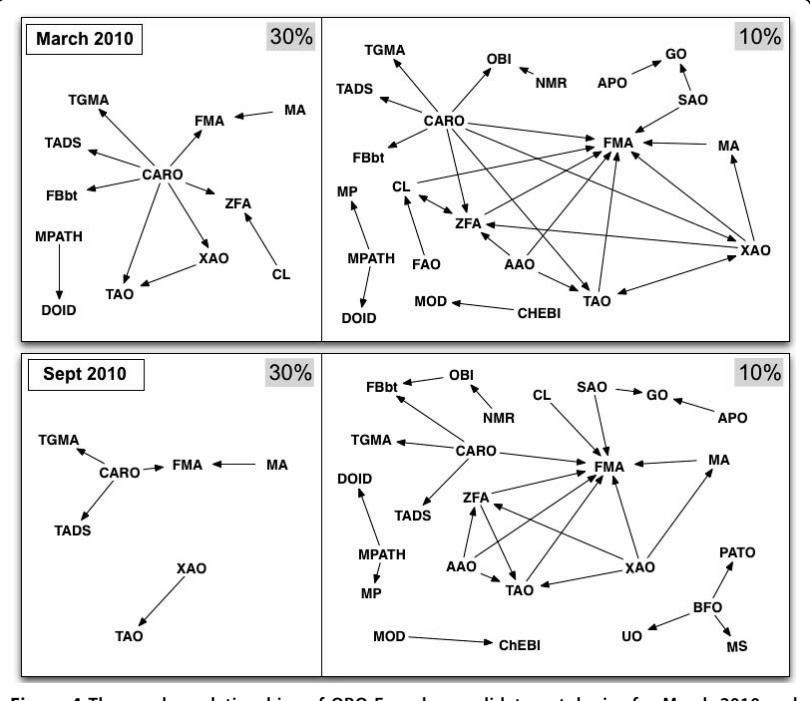
**The overlap relationships of OBO Foundry candidate ontologies for March 2010 and September 2010.** For each date, the two graphs show links between ontologies at 30% and 10% overlap. Nodes represent OBO Foundry candidate ontologies. An edge from a node representing an ontology *O*_1_ to a node representing an ontology *O*_2_ means that at least 30% (10%) of the terms in *O*_1_ map to some term in *O*_2_.

Figure 4 shows graphs representing the level of overlap between ontologies for March and September 2010. The left panel of each graph has connections between nodes representing ontologies if at least 30% of the terms in one ontology overlap with terms from the other ontology. The right panel of each figure represents the same graph, but with the edge created if 10% of the terms overlap. The figure shows that the number of ontology pairs where at least 30% of one ontology overlaps with the other decreased significantly between March and September 2010. Additionally, CARO had far fewer ontologies with which it shares a large portion of its terms.

We also identified new overlap introduced by ongoing ontology development. For example, as of September 2010, BFO overlaps with Units of Measurement (UO), Phenotypic Quality (PATO), and Mass Spectometry (MS), overlaps which did not exist in March 2010. BFO is a small, upper-level ontology designed to be reused for ontology building, particularly among OBO Foundry ontologies; however, UO, PATO, and MS do not explicitly reuse terms from BFO, but instead define new terms that correspond to the terms in BFO. Finally, we note that out of the 53 ontologies in our analysis, 17 ontologies (32%) had fewer than 4% of their terms overlapping with those of other ontologies and 32 ontologies (59%) had fewer than 10% of their terms overlapping with those of other ontologies. Furthermore, there are 7 ontologies (13%) with 5 or fewer total overlapping terms. By contrast, table 3 shows the ontologies that have the largest percent of their terms overlapping with terms from other ontologies in our latest snapshot.

**Table 3:** The amount of overlap for the OBO Foundry candidate ontologies.

Ontology	# of Overlapping Terms	Ontology Size (# of terms)	% of Overlapping Terms
CARO	48	48	100%
XAO	412	817	50%
MPATH	228	643	35%
MA	1,032	2,956	35%
SAO	268	821	33%
ZFA	815	2,593	31%
TAO	888	3,001	30%
BFO	10	39	26%
AAO	161	700	23%
APO	65	326	20%

Column (1) is the ontology id; (2) is the number of terms that overlap with terms from a different ontology; (3) is the ontology size; (4) is the percent of terms from the ontology that overlap with a term from another ontology.

### Reuse and overlap among the first designated OBO Foundry members

In our analysis of the six OBO Foundry members [5], we identified 179 cases of term reuse and 135 cases of term overlap. Most of the term reuse and overlap involves only two ontologies, Xenopus Anatomy and Development (XAO) and Zebrafish Anatomy and Development (ZFA). These two ontologies represent the anatomy and development of different organisms. Naturally, they share a large number of common terms, although those terms are not necessarily equivalent: They are anatomical parts of different organisms. The 179 terms that XAO reuses from ZFA are references through the *xref* field: An anatomical term describing xenopus in XAO references a term with the same name describing zebrafish in ZFA. However, the majority of overlapping terms in this set (109 of 135; 81%) are also between XAO and ZFA. The relationship of overlapping terms between these two ontologies is similar in nature to the relationship of the reused terms between them, identified through *xref*. Table 4 summarizes the remaining overlap among the six ontologies. Except for XAO and ZFA, no other pair of ontologies has more than 10 overlapping terms between them, but each ontology overlaps with at least one other ontology. The percentage of terms that overlap is significantly lower than the percentage that we observed among the candidates (Table 3).

**Table 4:** The amount of overlap for the six OBO Foundry members.

Ontology	# of Overlapping Terms	Ontology Size (# of terms)	% of Overlapping Terms
XAO	115	791	15%
ZFA	113	2,475	5%
PATO	4	1,263	0.3%
PRO	9	11,905	0.08%
GO	12	29,983	0.04%
CHEBI	8	24,225	0.03%

Column (1) is the ontology id; (2) is the number of terms that overlap with a term from another ontology; (3) is the ontology size; (4) is the percent of terms from the ontology that overlap with a term from another ontology.

## Discussion

We used a lexical matching algorithm to generate mappings because we have previously shown that this method has high precision and relatively good recall for biomedical ontologies. However, there are a few key limitations to our approach.

First, ontologies that are very small can strongly affect our analysis. For example, CARO and BFO both appear in our table of the ontologies with the largest percentage of overlap. BFO has more than 20% of its terms overlapping with terms from other ontologies, and, as we mentioned earlier, 100% of CARO terms overlap with terms in other ontologies. However, CARO contains only 46 terms and BFO contains only 39 terms so the large overlap percentages reflect only a handful of overlapping terms from these ontologies in reality.

Second, our analysis errs on the side of precision, with most mappings that we identify representing real overlap. Our analysis indicates that our recall is good, but not perfect. We previously found that, when identifying mappings between anatomy ontologies, recall for a lexical matching algorithm with less stringent requirements was 65%. Therefore, our more precise method most likely has recall below 65%. We found that lexical matching works relatively well for finding mappings between biomedical ontologies due to rich lexical information in these ontologies [6]; however, lexical methods cannot identify mappings between classes without lexical similarity. Since lexical matching identifies high-precision mappings with potentially limited recall, our numbers represent the lower bound on the number of overlapping terms. We need more advanced methods to identify the remaining overlap.

Finally, our analysis presents snapshots of the state of orthogonality at given points in time. The OBO Foundry is a work in progress as some of the OBO Foundry candidate ontologies are updated regularly. Therefore, the number of reused terms and the amount of overlap may change frequently.

Despite these limitations, our analysis allows ontology developers to form a more complete picture of inter-ontology relationships among OBO Foundry candidates and to understand how these relationships have evolved over time.

In the course of one year (September 2009 to September 2010), reuse among the 53 OBO Foundry candidates increased from 10,972 cases to 17,067 cases, with 13,358 cases of reuse at the half way point in March 2010. Additionally, about 63% of mapped terms reflect reuse of terms among OBO Foundry candidates in September 2010, compared to 53% in September 2009. Although both the absolute number of reuse cases and the percentage of mappings that represent reuse rather than overlap have increased, the percentage of OBO Foundry candidate terms involved in an overlap has remained relatively constant at around 3%. Thus, while reuse has increased in the OBO Foundry over the course of this year, overlap has remained relatively consistent both in terms of the absolute number of overlaps and in terms of the percentage of terms from the OBO Foundry that are involved in overlap.

Our results indicate that the nature of term reuse has evolved over time. We can see in Figure 2 that, from September 2009 to March 2010, most of the increased reuse resulted from increased use of the *xref* property to refer to classes, and from March 2010 to September 2010, most of the additional reuse consisted of terms using the same id, although the other two types of reuse increased as well.

Examining ontologies with significant reuse can also yield insights into the characteristics of those ontologies. For example, as of September 2010, OBI reuses terms from 10 different ontologies. OBI serves as a cross-disciplinary ontology for describing biological and clinical experimental processes and defines both broad terms that span multiple domains and domain-specific terms relevant to particular areas of study [14]. As such, OBI reuses terms from a number of different ontologies by design.

Although the authors of 16 ontologies reuse terms from other ontologies, this reuse exists in a variety of different forms. Indeed, there is currently no standard way for an ontology author to define that she is reusing a term from another ontology. In fact, a large portion of current reuse results from cases in which the id of one term is intended to reference another one explicitly, but does not match exactly.

By analyzing the dynamics of the overlap landscape (Figure 4), we can see how the distribution of overlaps among ontologies has changed over time. Although the level of overlap increased between March and September 2010, the number of ontology pairs for which at least 30% of one ontology overlaps with terms in the other ontology decreased. Additionally, the number of ontology pairs with at least 10% overlap decreased over the same period. This trend indicates that ontology development efforts have reduced some significant overlap among the ontologies, but a similar level of total overlap remains.

There are only 9 ontologies for which more than 20% of the terms overlap with terms in other ontologies. Additionally, there are only a handful of ontologies that have more than 10% of their terms mapped to any one other ontology. These facts, when coupled with the statistic that almost one-third of the ontologies have fewer than 4% of their terms overlapping with terms from other ontologies, suggest that there are a few ontologies with considerable overlap and many ontologies that each have just a small amount of overlap. Naturally, the larger the number of ontologies that require reconciliation with other ontologies, the more difficult the process: If two ontologies have 100 classes in common, the developers of only these two ontologies need to coordinate their efforts. If the same 100 overlapping terms are spread across 10 ontologies, ten teams of developers will need to be involved in the process of reconciliation, which will make the process more difficult.

Our results indicate that the largest amount of overlap is in the cluster of ontologies representing anatomy: the Foundational Model of Anatomy (FMA), Mouse Adult Gross Anatomy (MA), and ZFA each have more than 800 overlapping terms. At the same time, achieving orthogonality in this set may be rather difficult: The terms that the ontologies share describe different organisms, and therefore, one could argue they are not actually identical. However, in order to create a coherent set, *lung* in the MA, for example, must be related in some way to *Lung* in FMA. One possibility would be to create explicit mappings indicating homology and to keep the classes separate. Another possibility would be to create a naming convention to give organism-specific names to anatomical terms. For example, *lung* could be renamed to *mouse lung* in MA and *human lung* in FMA. Furthermore, every term in the Common Anatomy Reference Ontology (CARO) overlaps with at least one term from another ontology. CARO was designed as a reference ontology that will link together other anatomy ontologies, so it is not surprising to find significant overlap between CARO and the other anatomy ontologies. However, our results show that many anatomy ontologies do not reuse CARO terms explicitly.

Notably, our analysis does not account for the nature of the ontologies that we examine. For example, at the moment we do not distinguish between reference and application ontologies—a distinction that might give us further insight into the nature of the reuse and overlap among these ontologies. However, in order to account for the nature of the ontologies in our analysis, we would need to ensure that two prerequisites are met: First, we would require a clear set of criteria defining reference and application ontologies, and, second, we would need metadata from each ontology that would allow us to categorize it as a reference or application ontology.

Our results demonstrate that some overlap remains among the ontologies included as the first OBO Foundry members. Although there are only 135 overlapping terms among them, each of the six ontologies has some overlap and therefore a coordinated effort will be required to make these ontologies truly orthogonal.

The lack of any term reuse outside of the two anatomy ontologies in the set of the six OBO Foundry members indicates that this set of ontologies represents “low-hanging fruit” in achieving orthogonality: They represent disjoint domains and inclusion in the OBO Foundry did not require one ontology to drop some of its own terms and to reuse terms defined elsewhere. The reconciliation process will be more difficult for ontologies that represent overlapping domains when each defines a similar set of terms. Overall, among the 53 candidate ontologies that we analyzed, only 2 ontologies are orthogonal within the OBO Foundry, having no overlap with other candidates. Additionally, only 30% of ontologies reuse terms, while 96% have terms overlapping with other ontologies. Together, these statistics indicate that the vast majority of the ontologies that currently overlap have not yet adopted any measure of term reuse.

Our analysis indicates that several characteristics of the overlap among the ontologies will make reaching orthogonality difficult. First, many ontologies contain at least one term that overlaps with a term in another ontology, which means many developers will need to be involved in reconciling this overlap. Second, in some domains, such as anatomy, terms with closely related meanings may be difficult to reconcile. Finally, new ontology development can readily introduce new overlap among the ontologies. Given these overlap characteristics in combination with the fact that the number of overlaps among OBO Foundry ontologies did not decrease over the course of a year of active development, there is no evidence that the OBO Foundry will reach its stated goal of orthogonality, despite the increased adoption of term reuse among its ontologies.

## Conclusions

Our analysis produced a list of 9,992 pairs of overlapping classes that can serve as a guide for the developers of the OBO Foundry candidate ontologies in order to achieve orthogonality. We have created a prototype web application that allows OBO Foundry ontology developers to see which classes from their ontologies overlap with classes from other ontologies in the OBO Foundry (http://obomap.bioontology.org). We believe that there are several different categories for the pairs of overlapping terms identified by this analysis, each requiring a different development approach: (1) The terms represent the same concept; (2) The terms have the same name and represent related but different concepts (e.g., the anatomy example); and (3) The terms have the same name but are different terms, either because our analysis identified an incorrect match or because the terms are polysemous. Our results show that 51 ontologies (96%) of those listed on the OBO Foundry web site would require such attention. From our analysis, we conclude that while the OBO Foundry has made significant progress toward orthogonality during the period of this study through increased adoption of explicit term reuse, a large amount of overlap remains among these ontologies. Notably, the number of cases of term reuse increased by 6,095 terms over this period, from 4.0% of the total terms among the ontologies to 5.3% of the total. However, the total number of overlaps also increased during this period and 96% of the OBO Foundry candidate ontologies still have at least some overlap with the other ontologies. Additionally, some of the remaining overlap, such as that among the anatomy ontologies, requires careful consideration to reconcile. In this paper, we have shown that through analysis of the inter-ontology relationships among the OBO Foundry candidate ontologies, we can understand the current state of orthogonality in the OBO Foundry and how that state has evolved over time. Given that the overlap among the OBO Foundry candidate ontologies did not decrease from September 2009 to September 2010 and that this overlap remains spread across the vast majority of the ontologies, achieving orthogonality in the OBO Foundry will be an extraordinarily difficult task.

In future work, we plan to analyze where the overlap and reuse actually lie within the ontologies. Doing so might allow us to identify sub-domains of the ontologies that have either similar or conflicting representations. Such sub-domains would be important targets for reconciliation as they may reflect different perspectives or granularities with respect to the modeling of similar entities.

## Competing interests

The authors declare no competing interests.

## Authors’ contributions

AG collected the data, wrote the software, and performed the analysis. NN and MM participated in the design of the study and in drafting the manuscript. All authors read and approved the final manuscript.
